# A Novel 3D Culture System Using a Chitin-Based Polysaccharide Material Produces High-Quality Allogeneic Human UCMSCs with Dispersed Sphere Morphology

**DOI:** 10.3390/cells11060995

**Published:** 2022-03-15

**Authors:** Katsuhiko Kida, Tatsuro Kanaki, Shuang Gao, Daisuke Hatanaka, Masashi Iwakami, Shuai Liu, Masato Horikawa, Minoru Ono, Dehua Chang

**Affiliations:** 1 Biological Research Laboratories, Nissan Chemical Corporation, Shiraoka 349-0294, Saitama, Japan; kidak@nissanchem.co.jp (K.K.); iwakamim@nissanchem.co.jp (M.I.); horikawa@nissanchem.co.jp (M.H.); 2 BOE Regenerative Medicine Technology Co., Ltd., C2 Building, No.9 Jiuxianqiaobei Road, Chaoyang, Beijing 100015, China; 10143309@boe.com.cn (S.G.); lshuai@boe.com.cn (S.L.); 3 Materials Research Laboratories, Nissan Chemical Corporation, Funabashi 274-0052, Chiba, Japan; hatanakad@nissanchem.co.jp; 4 Department of Cardiac Surgery, University of Tokyo Hospital, 7-3-1 Hongo, Bunkyo-ku 113-8655, Tokyo, Japan; ono-tho@h.u-tokyo.ac.jp; 5 Department of Cell Therapy in Regenerative Medicine, University of Tokyo Hospital, 7-3-1 Hongo, Bunkyo-ku 113-8655, Tokyo, Japan

**Keywords:** human umbilical cord mesenchymal stem cells, 3D culture, allogeneic transplantation, paracrine factors, stemness

## Abstract

Mesenchymal stem cell (MSC) transplantation, in particular allogeneic transplantation, is a promising therapy for a variety of diseases. However, before performing allograft treatment it is necessary to find suitable donors, establish culture methods that maintain cell quality, and reduce cell production costs. Here, we present a new method of producing allogeneic MSCs combining human umbilical cord-derived mesenchymal stem cells (UCMSCs) and chitin-based polysaccharide fibers (Cellhesion^®^ MS). UCMSC numbers significantly increased, and cells grew as dispersed spheres on Cellhesion^®^ MS. Subsequent biological analyses showed that the expression levels of stemness-related and migration-related genes were significantly upregulated, including octamer-binding transcription factor 4 (OCT4), Nanog homeobox (NANOG), and C-X-C chemokine receptor type 4 (CXCR4). The secretion levels of paracrine factors such as prostaglandin E2 (PGE_2_), TNFα-stimulating gene (TSG)-6, fibroblast growth factor 2 (bFGF), and Angiogenin (Ang) from UCMSCs using Cellhesion^®^ MS were significantly higher than with microcarrier and U-bottom plate culture. In addition, culture supernatant from UCMSCs with Cellhesion^®^ MS had better angiogenic potential than that from monolayer cultured UCMSCs. Furthermore, we succeeded in a scaled-up culture of UCMSCs with Cellhesion^®^ MS using a closed culture bag. Therefore, Cellhesion^®^ MS is a key material for producing high-quality UCMSCs in a three-dimensional (3D) culture system.

## 1. Introduction

Mesenchymal stem cells (MSCs) isolated from various organs and tissues, such as bone marrow, adipose tissues, and umbilical cords, can be differentiated into adipocytes, chondrocytes, osteoblasts, cartilage, bone, skeletal muscle, and other cell types in specific conditions [1,2,3,4]. MSCs are applicable as treatment in a wide range of diseases (e.g., heart, immune, and inflammatory diseases), as based on their effects in immunomodulation, anti-inflammation, and angiogenesis. As a result clinical trials are underway [5,6]. MSCs secrete various paracrine factors, such as immunosuppressive factors, angiogenic factors, anti-apoptotic factors, and anti-oxidative factors involved in repair of damage of the endometrium and inhibition of the graft versus host response function [5]. Additionally, recent reports indicate that extracellular vesicles (EVs) present in MSC culture supernatants have a therapeutic effect on disease states [7,8]. Thus, MSC culture systems are capable of simultaneously producing two sources of therapeutics: the cells themselves, and culture supernatants containing EVs.

The two most common settings for stem cell transplantation are autologous and allogeneic. Although autologous MSC transplantation is the safest method, as there is no immune rejection, it may have some limitations in terms of time, cost, and quality [9]. Allogeneic MSC transplantation has attracted attention recently. One report suggested that the immunosuppressive properties and low immunogenicity of allogeneic MSCs effect a reduced immune response after transplantation [9]. Thus, allogeneic MSC transplantation have more potential and prospect in clinical application. It has also been reported that UCMSCs show higher OCT4 expression and have higher immunosuppressive effects than bone marrow-derived MSCs (BMMSCs) [10]. Therefore, UCMSCs are considered as a promising cell source for cell therapy.

For allogeneic MSC production, monolayer culture is the most conventional culture method. However, the spindle shape of cells observed in monolayer culture is not observed in vivo. When MSCs are cultured in monolayer conditions, the cell diameter increases with each passage, while the growth rate, stemness gene expression, and secretion of paracrine factors decrease [11,12,13]. Although a method for scaled-up MSC culture using microcarriers has been described, MSCs attached to carrier beads exhibit the same spindle shape observed in monolayer culture. In contrast, a suspension culture in stirring conditions which uses an ultra-low attachment plate and other 3D materials has produced MSCs with a round morphology [11]. However, MSCs undergo cell cycle arrest when cultured in these conditions, and cells cultured for extended periods undergo apoptosis-like anoikis due to the lack of scaffolding, which ultimately results in cell death [14]. To produce MSCs in 3D cell culture, it is essential not only to maintain quality but also to expand the MSCs.

Recently, we reported a 3D culture of UCMSCs using a chitin-based polysaccharide material [15]. This material enables EVs to be collected more efficiently, and EVs secreted from 3D cultured MSCs have higher immune-modulating potential than those from monolayer culture. However, pipetting was needed to culture MSCs with Cellhesion^®^ VP because of severe aggregation. Cellhesion^®^ MS is an insoluble chitin-based polysaccharide fiber. One unique characteristic of Cellhesion^®^ MS is that cells adhere to and proliferate on this material with a round shape, as in vivo. In addition, this material plays a role as a dispersant of MSC spheres. Therefore, aggregation is suppressed compared with Cellhesion^®^ VP.

In this study we evaluated the effect of a 3D culture using Cellhesion^®^ MS on the characteristics of UCMSCs. Significant up-regulation was observed in gene expression (OCT4, NANOG, and CXCR4) and paracrine factors (PGE_2_, TSG-6, bFGF, and Ang). In addition, Cellhesion^®^ MS enables the culture of UCMSCs with the suppression of large cell aggregation in large-scale conditions. Thus, this new 3D culture method is suitable for producing high-quality UCMSCs at large scale that are highly suitable for allogeneic stem cell transplantation.

## 2. Materials and Methods

### 2.1. Chemicals and Reagents

Cellhesion^®^ MS was obtained from Nissan Chemical Corporation. Human UCMSCs, Mesenchymal Stem Cell Growth Medium 2, human umbilical vein endothelial cells (HUVEC), Endothelial Cell Growth Medium, Endothelial Cell Basal Medium, DetachKit, PrimeScript™ RT Master Mix (Perfect Real Time), and Premix Ex Taq™ (Perfect Real Time) were purchased from Takara (Kusatsu, Shiga, Japan). MEMα with L-glutamine and Phenol Red was obtained from Fujifilm Wako Pure Chemical Corporation (Osaka, Osaka, Japan). The RNeasy mini kit was purchased from Qiagen (Valencia, CA, USA). All Taqman probes, Nunc MaxiSorp™ flat-bottom plates, FBS, nonessential amino acids, and BrdU staining kits were obtained from Thermo Fisher Scientific (Waltham, MA, USA). Recombinant human TNFα, human TSG-6 biotinylated antibody, and recombinant human TSG-6 were obtained from R&D Systems (Minneapolis, MN, USA). TSG-6 antibody was obtained from Santa Cruz Biotechnology (Dallas, TX, USA). Streptavidin (HRP) was purchased from Abcam (Trumpington, Cambridge, UK). TMB 1-Component Microwell Peroxidase Substrate, SureBlue, and TMB Stop Solution were obtained from SeraCare Life Sciences (Milford, MA, USA). The PGE_2_ ELISA kit was obtained from Enzo Life Sciences (Farmingdale, NY, USA). The bFGF ELISA kit was obtained from RayBiotech (Peachtree Corners, GA, USA). The Angiogenin ELISA kit was purchased from Proteintech (Rosemont, IL, USA). Culture bags were purchased from Nipro (Osaka, Osaka, Japan). Corning^®^ Matrigel^®^ Growth Factor Reduced (GFR) Basement Membrane Matrix, Phenol Red-free, LDEV-free, Costar^®^ 6-well and 24-well clear flat-bottomed ultra-low attachment well plates, Costar^®^ 24-well clear TC-treated well plates, α-MEM medium, RPMI 1640 medium, L-glutamine, 125 mL disposable spinner flasks, and Corning^®^ low-concentration Synthemax™ II microcarriers were obtained from Corning Incorporated (Corning, NY, USA). The CellTiter-Glo^®^ Luminescent Cell Viability Assay was obtained from Promega (Madison, WI, USA). Cell Counting Kit-8 and Cellstain^®^ (Calcein-AM) were obtained from Dojindo Laboratories (Kumamoto, Kumamoto, Japan). Anti-CD11b-PE (clone no. ICRF44), anti-CD19-FITC (clone no. HIB19), anti-CD34-PE (clone no. 581), anti-CD45-APC-cy7 (clone no. 2D1), anti-CD73-PE (clone no. AD2), anti-CD90-FITC (clone no. 5E10), anti-CD105-APC (clone no. 266), anti-HLA-DR-APC (clone no. G46-6), and stain buffer were obtained from BD Biosciences (Franklin Lakes, NJ, USA). Adipogenic, chondrogenic, and osteogenic differentiation kits were purchased from Cyagen (Santa Clara, CA, USA). Cell cycle kits were obtained from KeyGEN BioTECH (Nanjing, Jiangsu, China). PHA-L and Interleukin (IL)-2 were obtained from Sigma (Saint Louis, MO, USA). All other chemicals and solvents were of the highest grades that were commercially available.

### 2.2. Isolation of Umbilical Cord Mesenchymal Stromal Cells

Umbilical cord samples were collected from donors during childbirth. The criteria for selection of umbilical cord tissues were that they were from a full-term birth and were negative for HIV, HBV, HCV, HCMV, EBV, HTLV, HPV, and human parvovirus B19.

The umbilical cord samples were cut into 3 cm lengths and veins, arteries, and the tunica externa were removed. Next, the sample was washed three times with PBS, and cut into 1–2 mm^3^ fragments. The fragments were then uniformly placed in 100 mm diameter culture dishes with approximately 5 mm spacing. Finally, 4 mL of culture medium (α-MEM medium with 10% FBS, 1% nonessential amino acid, 1% L-glutamine, 4 ng/mL bFGF, and 40 unit/mL gentamicin) was added to each culture dish. The culture dishes were placed in an incubator at 37 °C, with 5% CO_2_ and 95% humidity. The culture medium was completely replaced every three days until cells migrated from the fragments and reached 70% confluence. The tissues were then removed, and the cells were passaged at a ratio of 1:4. Cells from the fourth passage were used in subsequent experiments.

### 2.3. Identity and Purity

UCMSC morphology was observed under an inverted microscope (Olympus CKX41; Shinjuku-ku, Tokyo, Japan) and images were captured with a camera (Canon EOS 700D; Ota-ku, Tokyo, Japan). Cellular phenotypes were evaluated for CD11b, CD19, CD34, CD45, CD73, CD90, CD105, and HLA-DR expression. The UCMSCs were washed twice in stain buffer prior to staining with anti-CD11b-PE, anti-CD19-FITC, anti-CD34-PE, anti-CD45-APC-cy7, anti-CD73-PE, anti-CD90-FITC, anti-CD105-APC, anti-HLA-DR-APC and isotype control antibodies for 30 min at room temperature and in the dark. The UCMSCs were then washed twice in stain buffer and re-suspended in 0.5 mL of stain buffer for flow cytometry. UCMSCs stained with isotype-APC, isotype-APC-cy7, isotype-FITC, and isotype-PE were used as respective controls.

UCMSCs can differentiate toward adipogenic cells, chondroblasts, and osteoblasts. Thus, adipogenic, chondrogenic, and osteogenic differentiation kits were used in accordance with the supplier’s specifications. Finally, adipogenic, chondrogenic, and osteogenic differentiation was evaluated by oil red O staining, alcian blue staining, and alizarin red staining, respectively.

### 2.4. Cell Cycle Analysis

The cell cycle was analyzed using a cell cycle kit. Briefly, 1 × 10^6^ UCMSCs were fixed in 1 mL of 70% ice-cold ethanol overnight. The fixed cells were then washed twice with PBS. Next, 100 µL of RNAse A was added and the cells were incubated at 37 °C for 30 min. After washing once with PBS, the fixed cells were suspended in 400 µL of PI staining solution for 30 min at 4 °C in the dark. Finally, the cell suspension was evaluated by flow cytometry.

### 2.5. Immunosuppressive Function

The immunosuppressive function of UCMSCs was evaluated by inhibiting peripheral blood mononuclear cells (PBMCs) proliferation, and a BrdU staining kit was used to label PBMCs and assess proliferation. UCMSCs were seeded into 6-well plates at a density of 5 × 10^4^ cells/cm^2^. When the UCMSC confluence reached 70%, PBMCs were seeded in the wells containing UCMSCs at a density of 1.25 × 10^5^ cells/cm^2^. 3 mL of RPMI 1640 medium containing 10% (*v*/*v*) FBS, 10 µg/mL of PHA-L, and 10 ng/mL of IL-2 was added to each well to activate PBMCs. The culture medium was changed two days later. On the fourth day, BrdU was added to the culture medium at a concentration of 10 µM. Cultivation of activated PBMCs alone was used as the positive control. Nonactivated PBMCs co-cultured with UCMSCs was used as the negative control. After 16 h, the PBMCs were collected and stained in accordance with the supplier’s instructions and evaluated by flow cytometry.

### 2.6. Human UCMSC Culture with Cellhesion^®^ MS

For monolayer culture, UCMSCs were cultured at passages 3 to 5. UCMSCs were seeded at a density of 15,000 cells/mL into Mesenchymal Stem Cell Growth Medium 2 containing 0.05% (*w*/*v*) of Cellhesion^®^ MS and dispensed into clear flat-bottomed ultra-low attachment 6-well plates or a culture bag (10 mL/well or 45 mL/culture bag). Half of the culture supernatant was removed and replaced with fresh medium every 3–4 days. The plates and culture bag were cultured unstirred for 1–2 weeks. Viable cells were counted by measurement of absorbance at 450 nm, using a SpectraMax^®^ 190 (Molecular Devices; San Jose, CA, USA) for the CCK-8 assay. For the ATP assay, an equal volume of ATP reagent (CellTiter-Glo^®^ Luminescent Cell Viability Assay) was added to the culture medium and the luminescent intensity (relative light unit (RLU) value) was measured using Enspire^TM^ (PerkinElmer; Waltham, MA, USA) at each time point. Cultured UCMSCs were stained by Cellstain^®^ at each time point.

### 2.7. Human UCMSC Culture with Microcarrier and in U Bottom Plates

UCMSCs were seeded at a density of 15,000 cells/mL into 45 mL of Mesenchymal Stem Cell Growth Medium 2 containing 400 mg of Corning^®^ low-concentration Synthemax™ II microcarriers and dispensed into a 125 mL spinner flask. For U-bottom plate culture, UCMSCs were seeded at a density of 15,000 cells/mL (200 µL/well). When using microcarriers, cell adhesion occurred in static conditions for 24 h after which cultures were agitated at 30 rpm for 15 min every three hours. Half of the culture supernatant was removed and replaced with fresh medium after four days.

### 2.8. Scanning Electron Microscopy (SEM) Evaluation

Sample preparation for scanning electron microscopy (SEM) was as follows. UCMSCs cultured with Cellhesion^®^ MS were fixed in 2% glutaraldehyde for three hours. The fixed UCMSCs and Cellhesion^®^ MS were then washed with PBS and immersed in ethanol for two hours, for dehydration. The resulting specimen was then washed with tert-butyl alcohol and applied to a glass plate. The specimen was then lyophilized, and Pt sputtering deposition was performed. SEM images were obtained using a Hitachi SU-8000 electron microscope (Hitachi, Ltd; Chiyoda-ku, Tokyo, Japan). The accelerated voltage of the electron beam was 1.7 kV.

### 2.9. RNA Isolation and Real-Time PCR

Total RNA was prepared using a RNeasy mini kit in accordance with the manufacturer’s protocols. The cDNA was synthesized from total RNA using PrimeScript™ RT Master Mix (Perfect Real Time). Real-time RT-PCR was performed using Premix Ex Taq™ (Perfect Real Time) with a 7500/7500 Fast Real-Time PCR System (Applied Biosystems; Waltham, MA, USA). The Taqman probes for NANOG, OCT4, C-X-C chemokine receptor type 4 (CXCR4), and glyceraldehyde-3-phosphate dehydrogenase (GAPDH) were Hs04399610_g1, Hs04260367_gH, Hs00607978_s1, and Hs99999905_m1, respectively. The PCR conditions for these genes were as follows: after an initial denaturation at 95 °C for 10 s, amplification was performed by denaturation at 95 °C for 10 s and annealing and extension at 60 °C for 34 s and 45 cycles. NANOG, OCT4, and CXCR4 mRNA levels were normalized to GAPDH mRNA.

### 2.10. Enzyme-Linked Immunosorbent Assay (ELISA) of PGE_2_, TSG-6, bFGF, and Angiogenin in Culture Supernatants

UCMSCs were seeded at a density of 50,000 cells/mL in Mesenchymal Stem Cell Growth Medium 2 containing 0.05% (*w*/*v*) of Cellhesion^®^ MS and dispensed into a clear flat-bottomed ultra-low attachment 24-well plate at 1 mL/well. For monolayer culture, UCMSCs were seeded at a density of 50,000 cells/mL in Mesenchymal Stem Cell Growth Medium 2 without Cellhesion^®^ MS and dispensed into a Costar^®^ clear TC-treated 24-well plate at 1 mL/well. These plates were cultured unstirred for 48 h, after which the cells were switched to fresh Mesenchymal Stem Cell Growth Medium 2 with or without 20 ng/mL recombinant human TNFα or MEMα with L-glutamine and phenol red containing 17% FBS, which is based on the previously reported conditions [16]. TNFα was treated to imitate the situation when inflammation was triggered. Cells were cultured for a further 24 h, after which the culture supernatants were harvested and viable cells were counted by measurement of absorbance or ATP assay, as described above. The quantification of PGE_2_, bFGF, and Angiogenin in culture supernatants was performed in accordance with the manufacturer’s protocols. The quantification of TSG-6 in culture supernatants was performed by ELISA and according to the conventional procedure, as follows: a Nunc MaxiSorp™ flat-bottomed ELISA plate was coated with a rat monoclonal antibody against hTSG-6. Human TSG-6 biotinylated antibody and streptavidin (HRP) were used for the primary and secondary detection antibodies, respectively. TMB 1-Component Microwell Peroxidase Substrate, SureBlue, and TMB Stop Solution were used for detection, and absorbance at 450 nm was measured using Enspire^TM^. TSG-6 concentrations were determined with a standard curve constructed by titrating rhTSG-6 (0–50 ng/mL).

### 2.11. Tube Formation Assay

For monolayer culture, HUVEC were cultured at passages 2–3. After the Matrigel^®^ was thawed, a pre-cooled 96 well plate was loaded with the Matrigel^®^ at 100 µL/well and placed in the culture incubator for 1 h. Then, HUVEC at a density of 2000 cells/50 µL Endothelial Cell Basal Medium/well were seeded in the 96 well plate and incubated with 50 µL conditioned medium harvested as above. After 4 h, the tubes were imaged using an inverted light microscope and quantified by the Angiogenesis Analyzer plug-in on ImageJ software.

### 2.12. Statistical Analyses

Statistical significance was analyzed with a Student’s *t*-test or Tukey’s test as indicated in each legend, using EXSAS version 7.1.6.1 (Arm Systex; Osaka, Osaka, Japan). The level of significance was set at 0.05 unless stated otherwise.

## 3. Results

### 3.1. Features of Cellhesion^®^ MS Culture

**Figure 1:**
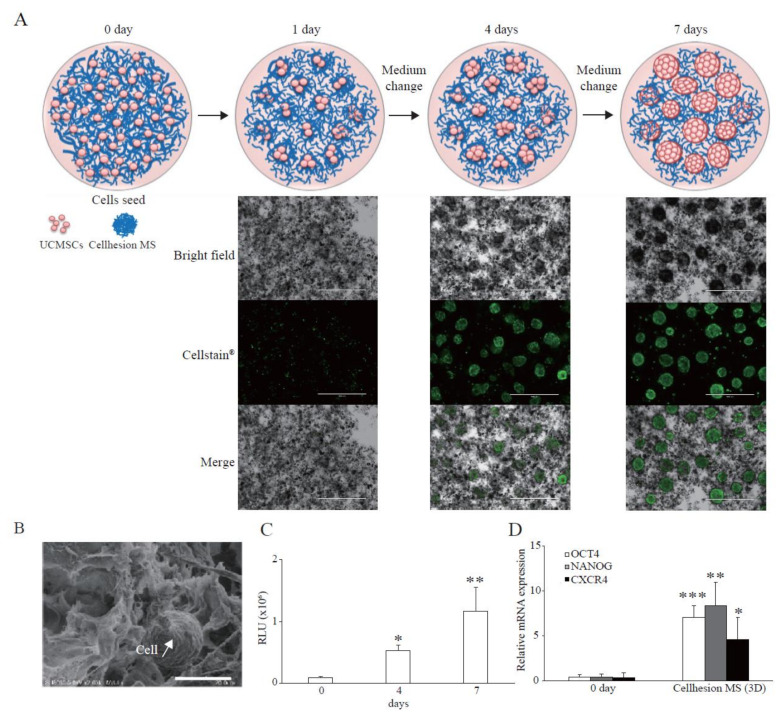
Features of Cellhesion^®^ MS culture. (**A**) Images of Cellhesion^®^ MS culture. Schematic representation of Cellhesion^®^ MS culture. Blue lines indicate Cellhesion^®^ MS and pink spheres indicate MSCs. UCMSCs were stained green by Cellstain^®^. Scale bar = 1000 μm. (**B**) SEM images of Cellhesion^®^ MS and UCMSCs. White arrows indicate UCMSCs. Scale bar = 20 μm. (**C**) The number of UCMSCs increased in a time-dependent manner. The number of UCMSCs was measured using an ATP assay. * *p* < 0.05, ** *p* < 0.01, compared with day zero. (**D**) The expression levels of NANOG, OCT4, and CXCR4 mRNA in UCMSCs increased. The expression levels of NANOG, OCT4, and CXCR4 mRNA in UCMSCs were determined by real-time RT-PCR and normalized to GAPDH mRNA levels. * *p* < 0.05, ** *p* < 0.01, *** *p* < 0.001 compared with day zero. Data show mean averages ± SD for three independent experiments.

To investigate the basic characteristics of Cellhesion^®^ MS culture, we analyzed the morphology of UCMSCs with Cellhesion^®^ MS using a microscope. When UCMSCs prepared in monolayer culture were seeded with Cellhesion^®^ MS, almost all cells adhered to Cellhesion^®^ MS within 24 h. When Cellhesion^®^ MS was in the medium under static conditions, it accumulated and formed weak aggregates; however, the weak aggregates were reversibly dispersed and induced to float with gentle stirring. We observed increased MSC cell–cell adhesion, proliferation, and formation of dispersed spheres with Cellhesion^®^ MS, which suppressed the formation of large cell aggregates in 3D culture condition. The size of spheres was around 300 μm (Figure 1A). A schematic representation of Cellhesion^®^ MS culture is also shown in Figure 1A. In addition, we analyzed Cellhesion^®^ MS and the morphology of UCMSCs with Cellhesion^®^ MS by SEM. Cellhesion^®^ MS exhibited a fiber-like structure in the medium (Figure 1B). Interestingly, the morphology of the attached MSCs remained round (Figure 1B). To confirm the number of UCMSCs increase, we evaluated the cell growth by ATP assay. The number of UCMSCs increased significantly by 5.8 times and 12.6 times at four and seven days (*p* < 0.05 and *p* < 0.01, respectively) (Figure 1C). Furthermore, to verify the effect of 3D culture using Cellhesion^®^ MS, we evaluated the expression levels of stemness-related genes such as OCT4 and NANOG and the migration-related gene, CXCR4. OCT4, NANOG, and CXCR4 mRNA expression levels in UCMSCs cultured with Cellhesion^®^ MS were significantly higher than UCMSCs at day zero (*p* < 0.001, *p* < 0.01, and *p* < 0.05, respectively) (Figure 1D). This result indicates that stemness- and migration-related genes in UCMSCs were upregulated in Cellhesion^®^ MS culture. Thus, UCMSCs could attach to and grow on Cellhesion^®^ MS, and the morphology of UCMSCs with Cellhesion^®^ MS differed from monolayer cultured MSCs. In addition, MSCs cultured with Cellhesion^®^ MS were able to gain enhanced stemness and migration ability.

### 3.2. Paracrine Factors from UCMSCs with Cellhesion^®^ MS

**Figure 2:**
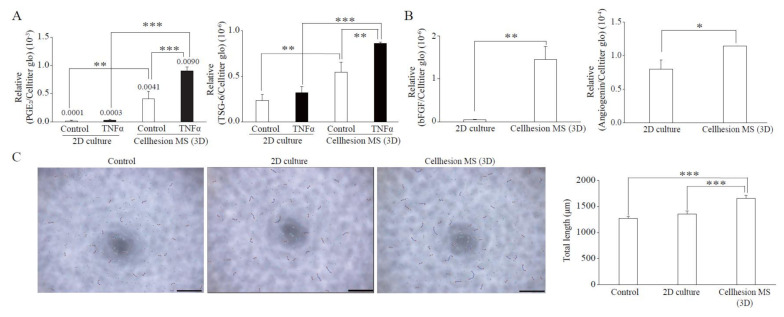
Paracrine factors from UCMSCs with Cellhesion^®^ MS. (**A**) The secretion levels of PGE_2_ and TSG-6 by UCMSCs cultured with Cellhesion^®^ MS increased, compared with monolayer culture. The secretion levels of PGE_2_ and TSG-6 by UCMSCs were measured by ELISA and normalized with an ATP assay. ** *p* < 0.01, *** *p* < 0.001, compared with each control or TNFα. (**B**) The secretion levels of bFGF and Angiogenin by UCMSCs cultured with Cellhesion^®^ MS increased, compared with monolayer culture. The secretion levels of bFGF, and Angiogenin by UCMSCs were measured by ELISA and normalized with an ATP assay. * *p* < 0.05, ** *p* < 0.01, compared with each culture. (**C**) The total length of HUVEC treated with CM from UCMSCs with Cellhesion^®^ MS was significantly longer than control and with CM from monolayer cultured UCMSCs. Total length of HUVEC in each condition was calculated by Angiogenesis Analyzer plug-in on ImageJ software. Each picture was one of the analyzed examples. Scale bar = 500 μm. *** *p* < 0.001, compared with control or CM from monolayer cultured UCMSCs. Data show mean averages ± SD for three independent experiments.

MSCs have immunomodulation, anti-inflammation, and angiogenesis effects resulting from secretion of paracrine factors. To verify the effects of 3D culture using Cellhesion^®^ MS, we evaluated typical paracrine factors from UCMSCs in each category compared with monolayer culture. We found that the secretion levels of PGE_2_ (immunomodulation) and TSG-6 (anti-inflammation) from UCMSCs with Cellhesion^®^ MS were significantly higher than from monolayer culture, whether with or without TNFα treatment (*p* < 0.001 and *p* < 0.01, respectively) (Figure 2A). Treatment with TNFα of UCMSCs with Cellhesion^®^ MS resulted in a significant induction of PGE_2_ and TSG-6 secretion (by 2.2 times and 1.6 times, respectively) (*p* < 0.001 and *p* < 0.01, respectively). In addition, the secretion levels of bFGF and Angiogenin (angiogenesis) from UCMSCs with Cellhesion^®^ MS were significantly higher than levels from cells in monolayer culture (*p* < 0.01 and *p* < 0.05, respectively) (Figure 2B). To evaluate the function of UCMSCs, the tube formation assay was performed using conditioned medium (CM) from each culture condition. The total length of HUVEC treated with CM from UCMSCs with Cellhesion^®^ MS was significantly longer than the control and CM from monolayer cultured UCMSCs (*p* < 0.001) (Figure 2C). Thus, MSCs cultured with Cellhesion^®^ MS were able to secrete far more paracrine factors, properties likely to affect treatment efficacy. 

### 3.3. Characterization of UCMSCs from Donors

**Figure 3:**
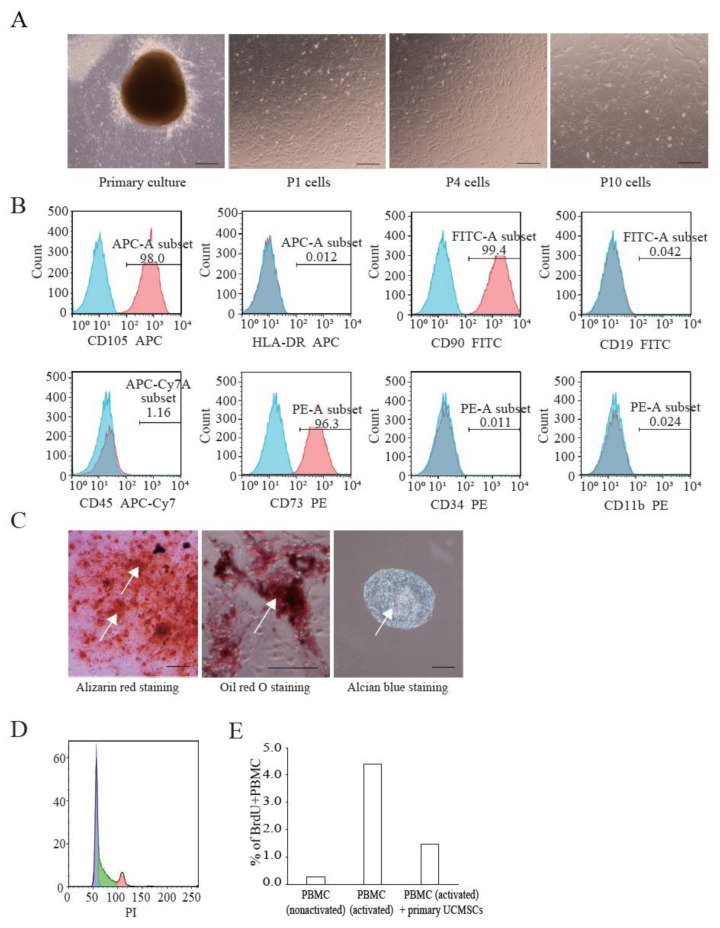
Characterization of UCMSCs from donors. (**A**) Culture and expansion of UCMSCs. Scale bar = 250 μm. (**B**) Representative flow cytometry histograms of human UCMSCs. UCMSCs were positive for CD105, CD90, and CD73, and negative for CD11b, CD19, CD34, CD45, and HLA-DR. Blue histograms are isotype controls and red histograms are experimental samples. (**C**) UCMSCs were successfully differentiated into adipogenic cells, chondroblasts, and osteoblasts. White arrows indicate stained cells. Scale bar = 500 μm. (**D**) Cell cycle analysis. (**E**) UCMSCs inhibit PBMC proliferation.

We established primary UCMSCs to apply Cellhesion^®^ MS culture. The umbilical cord samples used in this study were provided by donors at the time of childbirth. Initially, we investigated the basic characteristics of these established UCMSCs. Primary UCMSCs were usually observed 10–14 days after seeding tissue fragments. UCMSCs adhered well to the culture dish surface. P1 and P4 UCMSCs exhibited a fibroblast-like shape (Figure 3A). P10 UCMSCs exhibited a flat and elongated shape (Figure 3A). UCMSCs were positive for the common MSC markers: CD73 (96.3%), CD90 (99.4%), and CD105 (98%) (Figure 3B). UCMSCs were negative (or low) for the hematopoietic markers CD11b (0.024%), CD19 (0.042%), CD34 (0.011%), CD45 (1.14%), and HLA-DR (0.012%) (Figure 3B). UCMSCs were successfully differentiated into osteoblasts, adipocytes, and chondroblasts after 21 days of induction. The differentiated samples stained positive with alizarin red, oil red O, and alcian blue, respectively (Figure 3C). A cell cycle analysis indicated that 45.6% of UCMSCs were in the G0/G1 phase, 42.1% in the S phase, and 12.3% in the G2/M phase (Figure 3D). Immunosuppression studies indicated that UCMSCs effectively inhibited the proliferation of PBMCs. Results showed that the percent of new growing PBMCs reduced, from 4.39% to 1.46% during 16 h, when co-cultured with UCMSCs (Figure 3E).

### 3.4. Confirmation of Characteristics of Cellhesion^®^ MS Culture Using UCMSCs from Donors

**Figure 4:**
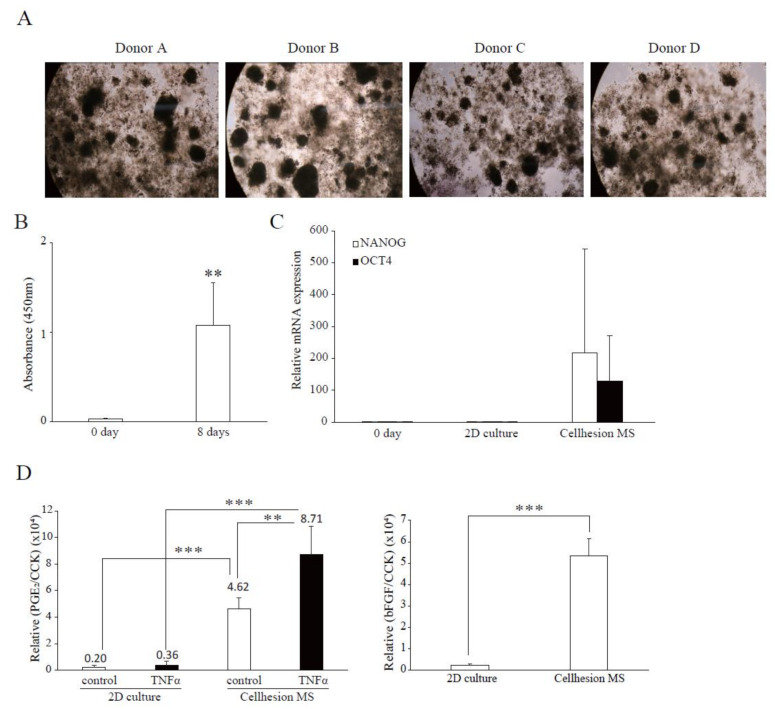
Confirmation of characteristics of Cellhesion^®^ MS culture, using UCMSCs from donors. (**A**) Images of cell morphology for each donor. (**B**) The number of primary UCMSCs from donors increased. The number of primary UCMSCs was measured using an ATP assay. ** *p* < 0.01, compared with day zero. (**C**) The expression levels of NANOG and OCT4 mRNA in UCMSCs on Cellhesion^®^ MS increased, compared with day zero and monolayer culture. The expression levels of NANOG and OCT4 mRNA in UCMSCs were determined by real-time RT-PCR and normalized to GAPDH mRNA levels. (**D**) The secretion levels of PGE_2_ and bFGF from UCMSCs cultured with Cellhesion^®^ MS increased, compared with monolayer culture. The secretion levels of PGE_2_ and bFGF from UCMSCs were measured by ELISA and normalized with a CCK assay. ** *p* < 0.01, *** *p* < 0.001, compared with control or TNFα. Data show mean averages ± SD for four independent donors.

To investigate differences between the four donors in terms of the effects of Cellhesion^®^ MS, and the difference between commercial UCMSCs and UCMSCs, we examined 3D cultures of four UCMSCs established from donor samples. In this culture, all primary UCMSCs formed spheres on Cellhesion^®^ MS (Figure 4A). Firstly, we evaluated the cell growth of UCMSCs; the number of UCMSCs significantly increased at eight days (*p* < 0.01) (Figure 4B). Next, we evaluated the expression levels of stemness-related genes and demonstrated that OCT4 and NANOG mRNA were upregulated, compared to day zero and a monolayer culture (Figure 4C). Finally, we evaluated paracrine factors from UCMSCs. The secretion level of PGE_2_ from primary UCMSCs with Cellhesion^®^ MS was significantly higher than monolayer cultured primary UCMSCs with TNFα (*p* < 0.001) and without TNFα (*p* < 0.01) (Figure 4D). The treatment of UCMSCs on Cellhesion^®^ MS with TNFα resulted in a significant (*p* < 0.01) induction of PGE_2_ secretion (by 1.9 times). The secretion levels of bFGF from UCMSCs with Cellhesion^®^ MS was significantly higher than monolayer cultured UCMSCs (*p* < 0.001) (Figure 4D). Thus, we obtained almost the same results for cell growth, gene expression, and paracrine factors with commercial and UCMSCs. These results suggest that this culture system could be suitable for any type of UCMSCs.

### 3.5. Scaled-Up Culture of UCMSCs Using a Culture Bag in a Closed Culture System

**Figure 5:**
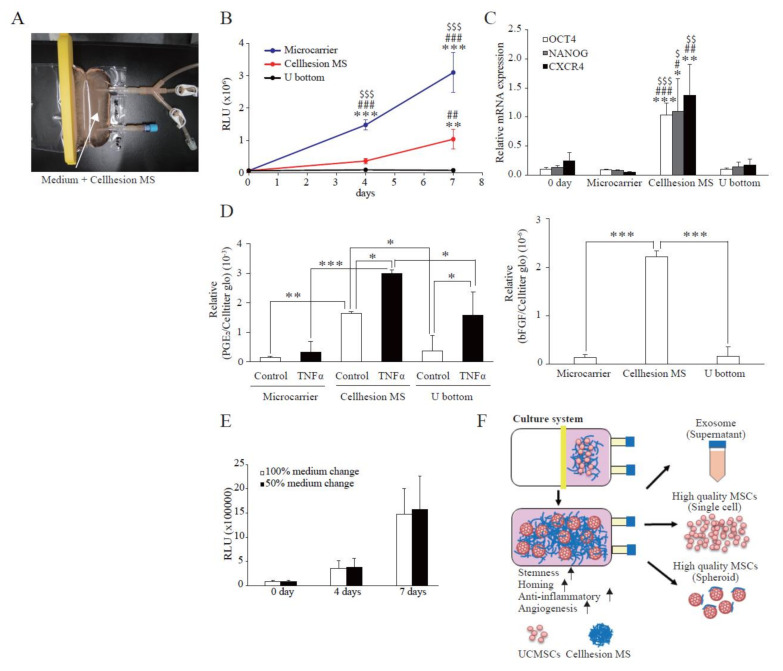
Scaled-up culture of UCMSCs using a culture bag to produce a closed culture system. (**A**) An image of the culture bag configuration. White arrows indicate medium containing 0.05% Cellhesion^®^ MS. (**B**) Comparison of cell growth in each culture method. The number of UCMSCs was measured using an ATP assay. ** *p* < 0.01, *** *p* < 0.001, compared with day zero. ^##^ *p* < 0.01, ^###^ *p* < 0.001, compared with U-bottom plate. ^$$$^ *p* < 0.001, compared with Cellhesion^®^ MS. (**C**) The expression levels of NANOG, OCT4, and CXCR4 in UCMSCs on Cellhesion^®^ MS increased, compared with microcarrier and U-bottom plate culture. The expression levels of NANOG, OCT4, and CXCR4 mRNA in UCMSCs were determined by real-time RT-PCR and normalized to GAPDH mRNA levels. * *p* < 0.05, ** *p* < 0.01, *** *p* < 0.001, compared with day zero. ^#^ *p* < 0.05, ^##^ *p* < 0.01, ^###^ *p* < 0.001, compared with microcarrier. ^$^ *p* < 0.05, ^$$^ *p* < 0.01, ^$$$^ *p* < 0.001, compared with U-bottom plate. (**D**) The secretion levels of PGE_2_ and bFGF from UCMSCs with Cellhesion^®^ MS increased, compared with microcarrier and U-bottom plate culture. The secretion levels of PGE_2_ and bFGF from UCMSCs were measured by ELISA and normalized with an ATP assay. * *p* < 0.05, ** *p* < 0.01, *** *p* < 0.001, compared with control or TNFα in microcarrier or U-bottom plate culture. (**E**) The number of UCMSCs was the same, for 50% and 100% volume medium replacement during the culture period. The number of UCMSCs was measured using an ATP assay in both conditions. (**F**) Schematic of a closed culture system using Cellhesion^®^ MS. Data show mean averages ± SD for three independent experiments.

To evaluate the possibility of large-scale UCMSC culture using Cellhesion^®^ MS, and for comparison with other culture methods, we attempted to culture UCMSCs with Cellhesion^®^ MS in a culture bag format (Figure 5A). Firstly, we evaluated the proliferation of UCMSCs with Cellhesion^®^ MS, microcarriers, and U-bottom plates. The cell growth of UCMSCs using microcarriers was significantly better than with Cellhesion^®^ MS and U-bottom plates at four and seven days (*p* < 0.001) (Figure 5B). From the viewpoint of spheroid culture, the cell growth of UCMSCs with Cellhesion^®^ MS was significantly better than with U-bottom plates at seven days (*p* < 0.01) (Figure 5B). Next, to verify the characteristics of UCMSCs on Cellhesion^®^ MS in a culture bag, we evaluated the expression of stemness-related genes (OCT4 and NANOG) and of CXCR4. The expression levels of OCT4, NANOG, and CXCR4 in UCMSCs with Cellhesion^®^ MS were significantly upregulated, compared with microcarrier and U-bottom plate culture (*p* < 0.05, *p* < 0.01 or *p* < 0.001, respectively) (Figure 5C). The secretion levels of PGE_2_ from UCMSCs with Cellhesion^®^ MS were significantly higher than in microcarrier and U-bottom plate culture, whether with TNFα (*p* < 0.001 and *p* < 0.05, respectively) or without TNFα (*p* < 0.01 and *p* < 0.05, respectively) (Figure 5D). The amount of bFGF secreted from UCMSCs with Cellhesion^®^ MS was significantly higher than with microcarrier and U-bottom plate culture (*p* < 0.001) (Figure 5D). Lastly, we investigated the effects of changes of medium volume on cell growth using Cellhesion^®^ MS. It was found that the UCMSC growth rate was the same, whether changing half the medium volume or carrying out full medium replacement (Figure 5E). These results suggest that an MSC culture using Cellhesion^®^ MS is a viable new culture system. This would facilitate large-scale culture of UCMSCs that gain stemness- and migration-related genes and secrete far more paracrine factors.

## 4. Discussion

Chitin is a long-chain polymer of N-acetylglucosamine, a derivative of glucose and a biodegradable bioadhesive and biocompatible material [17]. It has been approved by the U.S. Food and Drug Administration (FDA) for human use [18]. There are several reports about MSCs and chitin-based materials. One example is a deacetylated derivative of chitin: chitosan. Some researchers have reported that ADMSCs and placenta derived MSCs formed spheroids on chitosan, which is coated with hyaluronan, and that this material maintained OCT4, SOX2, and NANOG expression levels [19]. Other research demonstrated that the expression levels of CXCR4 were upregulated on a chitosan membrane [20]. In line with these reports, we investigated the effects of Cellhesion^®^ MS. UCMSCs formed spheres on Cellhesion^®^ MS and increased the expression of OCT4, NANOG, and CXCR4. In addition to the above reports, it has been reported that OCT4 and SOX2-overexpressing ADMSCs have a higher anti-inflammatory effect than control ADMSCs [21]. From this report it follows that the higher expression levels of OCT4 observed in UCMSCs with Cellhesion^®^ MS could affect the anti-inflammatory effect.

One benefit of using Cellhesion^®^ MS in MSCs 3D culture is affecting the features and quality of MSCs by enhancing cell–cell interactions. Traditional 3D-spheroid culture methods, such as the hanging drop and low attachment plate, can change MSC features such as stemness gene expression and secretion of paracrine factors [22]. In this study we observed spheres of UCMSCs on Cellhesion^®^ MS, similar to traditional spheroid culture methods. In accordance with previous reports, a Cellhesion^®^ MS-mediated 3D culture increases OCT4 and NANOG expression levels (Figure 1D, Figure 4C and Figure 5C) and paracrine factors (Figure 2A,B, Figure 4D and Figure 5D). In addition, unlike conventional 3D spheroid methods, Cellhesion^®^ MS culture enables UCMSCs not only to increase stemness-related genes and to secrete far more paracrine factors, but also to grow, as opposed to U-bottom plate culture (Figure 5B–D). The possible mechanism of this promotion may be due to the change of cellular morphology. There is report showed that MSCs in spheroids are more secretory than their monolayer counterparts [23]. The types of MSC 3D cultures that is biomaterials, also produce similar effects. The MSC in the middle of the spheroids and in the biomaterials, both experience limited adhesion and spreading, lack of polarization, less cellular strain due to less mechanical stiffness of the surroundings, a gradient of paracrine factors, and ultimately a common increase in mediators PGE_2_, hepatocyte growth factors. MSCs in spheroids had different mechanotransduction than the monolayer counterparts, which may regulate the interrelated signaling pathways, including RhoA/ROCK, Akt/Erk, or YAP/TAZ to affect the paracrine effect of MSCs [24]. We will investigate the detailed mechanism in further studies.

Another benefit of applying Cellhesion^®^ MS to large-scale high quality MSC culture is cost reduction. Some researchers have reported that it’s important to manufacture high-quality MSCs in massive volume and at lower costs to increase frequency of use [25]. For large-scale UCMSCs culture conditions, the cost of production space and labor account for large proportion. Cellhesion^®^ MS 3D cell culture method provides the possibility to expand UCMSCs in bioreactor tank, which could save space and reduce manual operation compared to 2D monolayer culture system, thus, 3D cell culture could save a lot of costs.

PGE_2_ is a bioactive lipid produced from arachidonic acid, with cyclooxygenase (COX) as the rate-limiting enzyme. PGE_2_ is an immunosuppressive factor secreted by MSCs. By reducing the activation and proliferation of NK and T cells, it is possible to realize not only GVHD treatment but also allogeneic transplantation [10,26]. UCMSCs exhibit a higher expression of the OCT4 gene than BMMSCs, but also have a greater ability to suppress the proliferation of T cells involved in inflammation [10]. PGE_2_ is an essential factor in the suppression of T cell proliferation related to inflammation, suggesting that it is better to use UCMSCs for allogeneic transplantation than BMMSCs [10]. Furthermore, it has been reported that PGE_2_ plays a prominent role in the mechanism of various immunomodulatory factors [10] and has an important role in therapy for various diseases. It has been reported that MSCs administered to GVHD model mice show therapeutic effects, with PGE_2_ being one of the main therapeutic mediators [27]. In addition, it has been reported that PGE_2_ is a marker for predicting therapeutic effects in the treatment of traumatic brain injury with MSCs [28]. PGE_2_ is also known to promote the migration of MSCs via FAK and ERK1/2 [29] and may increase the efficiency of its own migration. The combination of UCMSCs and 3D culture using Cellhesion^®^ MS is the most suitable method of producing allogeneic MSCs, because of their ability to secrete high levels of PGE_2_.TSG-6 is a representative secretory factor that exhibits immunosuppressive activity and has been reported to be a factor in the therapeutic effect of MSCs on various diseases [30]. It has been reported that when human BMMSCs are administered to acute pancreatitis model mice, TSG-6 acts as a major secretory factor and exhibits a therapeutic effect [31]. In addition, it has been reported that human BMMSCs produce a therapeutic effect via TSG-6 in lipopolysaccharide-induced lung injury model mice [32]. The amount of PGE_2,_ TSG-6, bFGF and Angiogenin secreted from MSCs cultured with Cellhesion^®^ MS is higher than in MSCs in monolayer culture, indicating that a Cellhesion^®^ MS culture is more efficient in producing cells to treat diseases such as ischemic heart disease and cartilage injury [33,34]. Besides that, the 3D culture methods also tries to reduce the gap between in vitro and in vivo drug testing models.

Large-scale MSC culture has been used to produce not only MSCs but also EVs (from culture supernatants) as therapeutics [7,8]. The medium used for culturing MSCs typically contains bFGF [35]. bFGF is an important factor for promoting MSC proliferation and the maintenance of stemness [36]. However, it is expensive and must be continually added to the culture medium because of its rapid degradation [37]. Therefore, the cost of bFGF is an important component of the total cost of MSC production. Recently, low molecular weight compounds that exhibit bFGF-like effects have been screened [38]. In the culture system using Cellhesion^®^ MS, MSCs secrete bFGF at a practical level; it is likely that the amount of medium exchange can be considerably reduced with Cellhesion^®^ MS. Cell growths with 50% volume media changes and with 100% medium changes gave the same results (Figure 5E). This has great merit in terms of reducing the cost of medium in MSC production, compared with monolayer culture and other culture methods. Thus, our culture method can also be applied to EV production, which enables a scaled-up culture and reduced culture costs.

Limitation still existed in this study, for example, there was sample difference. In this study, we used commercial UCMSCs to establish the Cellhesion^®^ MS culture system. Then, UCMSCs from donors were used to verify this 3D culture system, because UC-MSCs from donors will be used in the clinical cell therapy and there will be individual difference. Our experiment results (Section 3.4) showed that UCMSCs from donors could be cultured and expanded on Cellhesion^®^ MS. Compared to 2D monolayer culture, UCMSCs culture on Cellhesion^®^ MS had higher NANOG, OCT4 gene expression and PGE_2_, bFGF secretion. This demonstrated the 3D culture system based on Cellhesion^®^ MS has general applicability. The results also demonstrated that it is necessary to screen UCMSCs with higher viability to achieve better clinical effect. In the future, we will test more samples from donors to establish a UCMSCs screening criteria according to big data of quality study, which will provide UCMSCs seeds for scale-up cultivation in consistent quality. Besides that, we will construct a closed culture system for UCMSCs using Cellhesion^®^ MS (Figure 5F). The open style of monolayer culture methods complicates the production of cells, which increases the cost of quality testing and limits the production equipment to prevent cross-contamination. Our study suggests that high-quality UCMSCs can be cultured by combining oxygen-permeable bags and Cellhesion^®^ MS in a closed system culture model.

## 5. Conclusions

The 3D culture method using Cellhesion^®^ MS enables UCMSCs to secrete high levels of immunosuppressive factors, suggesting the possibility of producing MSCs with superior allogeneic properties. In addition, cells grown using this culture method were able to gain stemness expression, unlike other culture methods, and to produce the high levels of bFGF secretion necessary for MSC proliferation. It is expected that this culture method may become an important technology in the development of a closed culture system.

## Data Availability

The data that support the findings of this study are available from the corresponding authors, Tatsuro Kanaki, and Dehua Chang, upon reasonable request.
